# Contribution of Genomics to the Surgical Management and Study of Oral Cancer

**DOI:** 10.1245/s10434-021-09904-0

**Published:** 2021-04-12

**Authors:** Zuzana Saidak, Claire Lailler, Sylvie Testelin, Bruno Chauffert, Florian Clatot, Antoine Galmiche

**Affiliations:** 1 UR7516 “CHIMERE, Université de Picardie Jules Verne”, Amiens, France; 2 Centre de Biologie Humaine, CHU Amiens, Amiens, France; 3 Department of Maxillofacial Surgery, CHU Amiens, Amiens, France; 4 Department of Oncology, CHU Amiens, Amiens, France; 5 Centre Henri Becquerel, Rouen, France; 6 INSERM U1245/Team IRON, Rouen, France

## Abstract

**Background:**

Oral squamous cell carcinoma (OSCC) is the most frequent type of tumor arising from the oral cavity. Surgery is the cornerstone of the treatment of these cancers. Tumor biology has long been overlooked as an important contributor to the outcome of surgical procedures, but recent studies are challenging this concept. Molecular analyses of tumor DNA or RNA provide a rich source of information about the biology of OSCC.

**Methods:**

We searched for relevant articles using PubMed. We examined in particular the prospect of applying molecular methods for minimally invasive exploration of OSCC biology.

**Results:**

We examined five potential applications of genomics to the surgical management and study of OSCC: i) assessing oral potentially malignant lesions; ii) tumor staging prior to surgery; iii) predicting postoperative risk in locally advanced tumors; iv) measuring minimal residual disease and optimizing the longitudinal monitoring of OSCC; and v) predicting the efficacy of medical treatment.

**Conclusions:**

Genomic information can be harnessed in order to identify new biomarkers that could improve the staging, choice of therapy and management of OSCC. The identification of new biomarkers is awaited for better personalization of the surgical treatment of OSCC.

The oral cavity (lips, buccal mucosa, anterior tongue, hard palate, floor of mouth, and alveolar ridge) constitutes the most frequent primary location of head and neck squamous cell carcinoma (HNSCC).^1^,^2^ Oral squamous cell carcinoma (OSCC) classically occurs in a context of chronic alcohol and tobacco use.^1^,^2^ Areca nut-chewing also constitutes an established risk factor for OSCC in specific areas of Asia.^2^

Although human papillomavirus (HPV) has not been established as having a role in the development of OSCC, the oral cavity is characterized by the presence of an oral microbiote, whose contribution to the induction of inflammation and tumor promotion in this location is discussed.^3^ Management of OSCC involves different medical specialties with complementary expertise, but surgery usually is the first treatment method. Tumor anatomic determinants are in general the main criteria used for the initial prognosis assessment. The final pathologic examination of the tumor establishes the choice of therapy.

The recent revised version of tumor-node-metastasis (TNM) recognizes the increasing importance of features related to tumor biology. For example, the depth of invasion (DOI) is a useful parameter, together with the well-known criteria that determine OSCC prognosis including the presence of invaded surgical margins (SMs), extra-nodal extension (ENE), lymphovascular invasion (LVI), and perineural invasion (PNI).^4^ Positive SM or ENE identify advanced tumor stages that require heavy medical treatments. Both LVI and PNI play an important role in the choice of treatment for early OSCC, although the corresponding pathologic diagnoses might sometimes be difficult and poorly standardized.^5^ The need to introduce biomarkers and standardize medical practices is fueling a growing interest in tumor biology as a determinant of the OSCC response to surgery.

Oral oncogenesis is driven by the accumulation of genomic alterations in the epithelial cells of the oral cavity^6^–^8^(Table 1). The most common driver mutations found in OSCC occur in the *TP53* gene. These mutations occur at an early stage during oral carcinogenesis, possibly in a single adult stem cell. Replacement of the normal epithelium by cellular clonal units prone to accumulation of further genomic alterations accounts for the later steps of malignant transformation.^9^

**Table 1:** A summary of the most common genomic mutations/CNA found in OSCC^a^

Gene	Protein/function	Type of alteration (%)
*CDKN2A*	p16/cell cycle control	**Mutations (26%), deep deletion (33%)**
*TP53*	p53/cell cycle control	**Mutations (76%),** deep deletion (1.3%)
*CCND1*	cyclin D1/cell cycle control	**Amplifications (22%)**
*EGFR*	EGF receptor/growth regulation and oncogenic-signaling	Mutations (5%), **amplifications (12%)**
*PIK3CA*	PI3K catalytic subunit/growth regulation and oncogenic-signaling	**Mutations (17%), amplifications (14%)**
*PTEN*	PTEN/growth regulation and oncogenic-signaling	Mutations (1.9%)
*FAT1*	FAT1 protocadherin/growth regulation and oncogenic-signaling	**Mutations (27%)**, deep deletion (7%)
*AJUBA*	AJUBA/growth regulation and oncogenic-signaling	Mutations (6%)
*NOTCH1*	NOTCH1/growth regulation and oncogenic-signaling	**Mutations (21%),** deep deletion (2.9%)
*KMT2D*	KMT2D histone methyl-transferase/regulation of epigenetic marks	**Mutations (13%)**
*NSD1*	NSD1 histone methyl-transferase/regulation of epigenetic marks	Mutations (8%)

CNA, copy number alteration; OSCC, oral squamous cell carcinoma

^a^The list is restricted to 11 biologically significant genes with reported functions as oncogenes and anti-oncogenes. The  % of genomic alterations in OSSC was calculated based on data retrieved from The Cancer Genome Atlas (TCGA) (*n* = 321 OSCC). The table shows the  % of tumors with mutations or CNAs (amplifications or deep deletions) in selected genes. Note that only alterations found in more than 1% of tumors are shown. Alterations detected in more than 10% of OSCC are in bold

Genomic analyses of tumor DNA/RNA provide a rich source of information regarding tumor biology and can be performed using a large variety of protocols and platforms. The variety of analytical strategies offers tailored opportunities to explore tumor biology but represents a challenge in the transposition of genomic research to the clinical setting.^10^,^11^ The number of pre-analytical parameters (e.g., biopsies vs surgically resected samples, frozen vs formalin-fixed specimens, perioperative hypoxia) is a source of variability between studies.

Amplification by polymerase chain reaction (PCR) typically is used as an initial step to amplify DNA and to analyze the sequence or expression of a panel of genes. Microarrays and assays that rely on DNA hybridization, such as the NanoString platform (bar coding of nucleic acids) permit large-scale analysis of DNA and RNA expression profiling. Overall, however, the fast pace of technological progress has been illustrated by massively parallel sequencing (the so-called next-generation sequencing [NGS]).^10^,^11^

The most classical applications of tumor DNA analysis include detection of DNA mutations, copy number alterations (CNA), and the presence of structural modifications in the tumor genome. Functional genomics (i.e., the study of mRNA levels, translated into gene expression signatures) also has gained popularity because it offers opportunities for integrative studies covering virtually any facet of tumor biology^10^,^11^ (Fig. 1).

**Fig. 1:**
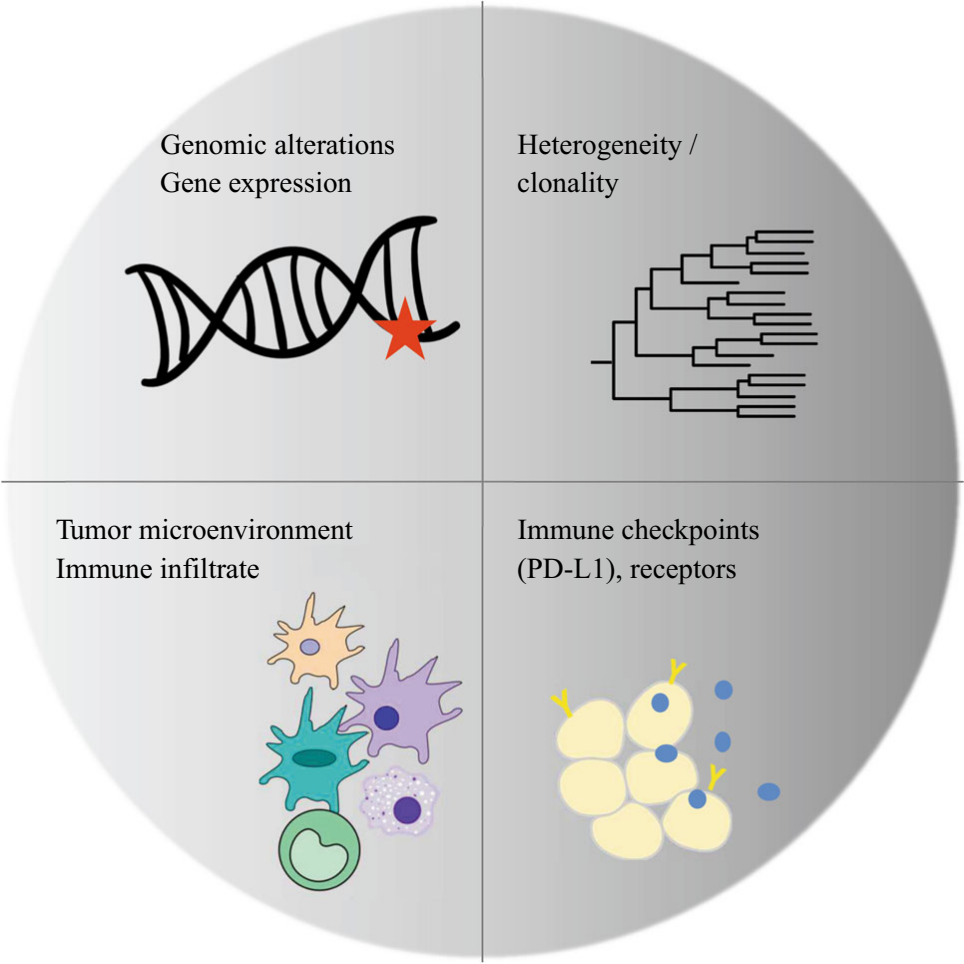
An overview of the information that can be gained from tumor genomic analysis. DNA/RNA sequencing from tumor material permits identification of genomic mutations and structural rearrangements that define the mutational burden of a tumor, some of which can be targeted therapeutically. Analyzing tumor gene expression also provides information regarding the functional status of the tumor (e.g., the presence of hypoxic areas). Recent practical strategies permit analysis of an individual tumor’s clonal structure and the reconstruction of its evolution in a dynamic fashion, potentially providing useful information regarding its response to treatment. Another important aspect is analysis of the composition of the tumor microenvironment (TME), including its infiltration with immune cells. The density of T cell infiltrate, the functionality of T cells, and the immune receptor repertoire can be assessed directly through functional genomics

More recent applications of functional genomics include the study of the tumor microenvironment^12^ and single-cell analyses, increasing the resolution of genomic analyses for the study of cellular heterogeneity within an individual tumor.^13^ Importantly, the revolution of open access data pioneered by The Cancer Genome Atlas (TCGA), a database offering easy access to semi-processed genomic data of tumors, facilitates the confrontation of clinical questioning and genomic analysis. This review discusses the practical applications of genomics in the management and study of OSCC.

## Assessing the Risk Associated with Oral Potentially Malignant Lesions

Oral potentially malignant lesions (OPMLs) are heterogeneous and include leukoplakia, erythroplakia, dysplastic lesions. The OPMLs carry a moderate yet significant risk of malignant transformation, estimated to be approximately 2% to 5% during a lifetime.^14^ Their pathologic diagnosis is difficult because of the multiple cytologic alterations that can be found (e.g., altered epithelial stratification, loss of epithelial polarity, nuclear dysmorphia, dyskeratosis), reflecting the complex biology of tumor development in the oral cavity. It also can be difficult to distinguish between OPMLs and inflamed and regenerative epithelia, which commonly occur in the oral cavity. These difficulties, combined with variability in sampling, explain why the diagnosis is poorly standardized and why the inter-observer agreement is incomplete.^14^

Because of the medical burden that OPMLs represent, a great need exists for biomarkers to identify the lesions that require surgical treatment.^14^ Loss of heterozygosity (LOH) currently is the most predictive marker of the malignant potential of OPMLs. Typically, LOH is analyzed using microsatellite loci because these short repeated sequences present with a high frequency of polymorphisms. In the prospective study by Zhang et al.,^15^ simultaneous LOH of chromosomal regions 3p14 and 9p21 was reported to be associated with more than a 22-fold increased risk for 5-year progression of mild to moderate dysplasia to cancer. The results of this study and others established that OPMLs already exhibit some of the genomic alterations found in OSCC. Although their detection might help to standardize the pathologic diagnosis of malignancy, a key challenge remains to establish minimally invasive strategies applicable to the potentially extensive areas of oral mucosa that need to be screened for “dangerous” lesions eligible for surgical resection.

The study by Graveland et al.^16^ examined the possibility of simultaneously detecting common LOH and missense mutations of *TP53* in oral swirls from patients with leukoplakia. More recently, Foy et al.^17^ proposed expanding the focus of genomic analyses of OPML by further classifying leukoplakia based on gene expression and the presence of an mRNA profile enriched with genes related to lymphocyte and monocyte function. Analyzing gene expression to define OPML with immunoactive profiles not only might be important to refining the prediction of the malignant risk^18^ but also might be useful in the consideration of alternative immunotherapeutic protocols.^19^

MicroRNAs are short non-coding single-strand RNAs (usually 19 to 25 nucleotides) that control gene expression at the post-transcriptional level, typically by interacting with the 3′-untranslated region of their target mRNA.^20^ The recent comprehensive exploration of the miRNome by NGS showed that specific expression profile alterations exist in OSCC.^21^ Importantly, miRNAs also are found in extracellular vesicles that protect them from nucleolytic attack and increase their stability in biological fluids, making them potential biomarkers in plasma or saliva (either as apoptotic bodies, microvesicles produced by membrane budding, or exosomes originating from the endosomal apparatus).^22^,^23^

A study by MacLellan et al.^24^ described the utility of analyzing the serum levels of miRNA in predicting the malignant potential of high-risk oral lesions (OSCC and dysplastic lesions). A more recent study by Yap et al.^25^ used quantitative PCR (qPCR) to profile the expression of five miRNAs from oral rinses in a cohort of patients with OSCC and OPML. This profiling was able to differentiate robustly between OPML and OSCC (sensitivity, 86.8%; specificity, 81.5%).^21^

The interesting prospect of testing salivary miRNA was explored by Romani et al.,^26^ who reported that large amounts of one miRNA (miR-432-5p) were detected in the saliva of OSCC patients compared with healthy controls. A combination of three miRNAs analyzed in the saliva was able to robustly diagnose OSCC (area under the curve [AUC], 0.98).^26^ Because of its noninvasive nature, profiling miRNA in saliva offers promising perspectives as a way to assist the biological diagnosis of OSCC. The role of salivary miRNA analyses needs to be addressed in future prospective studies.

**Fig. 2:**
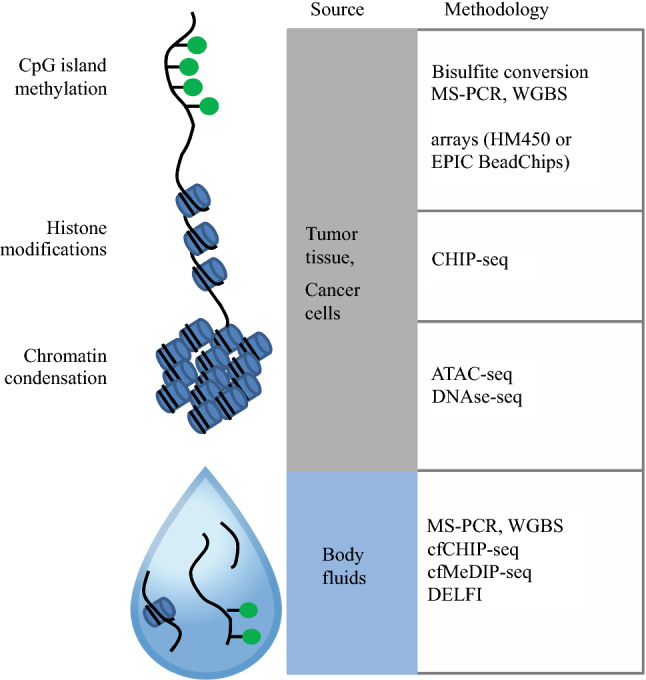
DNA methylation and histone modifications as epigenetic marks and the corresponding methodologic approaches. The DNA methylation of CpG dinucleotides represses transcriptional activity. Chromatin condensation, typically regulated by post-translational modifications of histones, is another important determinant of gene expression. Various analytical strategies allow for targeted or genome-wide analyses of epigenetic marks. A common strategy used to analyze DNA methylation relies on DNA conversion by sodium bisulfite. Unmethylated cytosine (but not its methylated counterpart) is converted to uracil, which is recognized as thymine in subsequent reactions. Amplification by PCR and sequencing then can be used to perform targeted or genome-wide analyses of DNA methylation (methylation-specific PCR assay [MS-PCR] and whole-genome bisulfite sequencing [WGBS]). Array-based technologies constitute an accessible technique for genome-wide methylation analyses and have been used in The Cancer Genome Atlas (TCGA) (HM450). Chromatin immunoprecipitation with DNA sequencing (CHIP-seq) can be used to explore the post-translational modifications of histones. To explore chromatin accessibility for research purposes in cells and tissues, DNAse-seq (DNase I hypersensitive sites sequencing) and ATAC-seq (assay for transposase-accessible chromatin) approaches can be used. A recent development is the possibility of analyzing epigenetic marks in body fluids using cell-free DNA (cfDNA). Most studies to date perform targeted analyses using MS-PCR to analyze DNA methylation of cfDNA from either serum or saliva. A smaller number of studies recently have reported the use of the cfMeDIP-seq or ChIP-Seq strategies using the serum of cancer patients. Another promising strategy based on measuring the size of cfDNA fragments in the serum (DNA evaluation of fragments for early interception [DELFI]) was recently reported. This strategy is based on low-coverage sequencing of the cfDNA released by cancer cells in the blood because DNA packaging modulates the sensitivity of the genome to fragmentation. These new approaches offer the exciting prospect of noninvasive genome-wide exploration of tumor epigenetics, but their use has not been reported in oral squamous cell carcinoma (OSCC)

DNA methylation is a potent and ubiquitous epigenetic modality^27^ (Fig. 2). In general, DNA methylation of cytosine-guanine dinucleotides (CpG) reduces the transcriptional activity of the methylated promoter. The genome of OSCC cells is characterized by a global reduction of CpG methylation and local spots of hyper-methylation.^27^ Because CpG methylation contributes to the silencing of some important tumor suppressor genes, such as *CDKN2A*, it is a likely motor event during oral carcinogenesis.^27^

The study of epigenetics is important for two reasons. First, because epigenetics is a biological link to the environment, nutrition, the inflammatory context, and the oral microbiote may contribute to the malignant transformation by altering epigenetic marks.^28^ Second, epigenetics is a source of potential biomarkers for cancer diagnosis.^29^ Figure 2 shows the main epigenetic marks and some of the powerful techniques that are applicable in the clinical setting. The study of the DNA methylome of OSCC and OPML has established a landmark for the application of this type of analysis to the early stages of oral carcinogenesis.^30^–^32^

Epigenetic analyses can be performed in OSCC noninvasively by oral brushing, mouth rinsing, or saliva samples.^33^ Multiple studies have examined the possibility of discriminating OSCC from healthy oral mucosa and non-malignant lesions using this type of samples and methylation-specific PCR (MS-PCR) analysis to target 3 to 13 methylated genomic loci.^34^–^38^

In parallel, the emergence of multi-cancer early detection (MCED) blood tests based on cell-free DNA (cfDNA) analysis is an important development.^39^ The large-scale analyses of epigenetic marks from cfDNA in the serum could significantly help in early diagnosis of cancer.^40^,^41^ The recent study by Liu et al.,^41^ based on the analysis of more than 1 million CpGs and more than 10^5^ methylated regions, examined the sensitivity and accuracy of this strategy in diagnosing more than50 cancer types, including HNSCC. Due to the ubiquitous nature of DNA methylation and its strong link to cell differentiation, DNA methylation detection might be more sensitive and accurate in diagnosing early cancer than mutational/CNA analysis.^41^

No data exist regarding the applicability of MCED tests to OPML. Nevertheless, MCED assays are raising considerable interest as an adjunct to population-based screening for the most common cancers.^39^ Prospective trials to examine the utility of MCED in clinical practice are required.

Importantly, research in epigenetics is a burgeoning field, with several studies and recent technical developments (e.g., the study of cfDNA fragmentation).^42^ The coming years promise to bring a deeper understanding concerning the extent of epigenetic alterations in OSCC and OPML. This knowledge could pave the way to a better, noninvasive biological diagnosis of oral cancer, with practical implications in the monitoring of OPML and in defining of surgical indications.

## Staging of OSCC Before Surgery

Early-stage disease (i.e., small tumors without prominent extension to lymph nodes) represent about 30% to 40% of all tumors of the oral cavity.^1^ Oral squamous cell carcinomas have a recognized propensity to form node micrometastases that can be missed by imaging.^1^ Accurately staging the cN0 OSCC is essential in order to avoid the need of using heavy adjuvant radio(chemo)therapy after an initially planned curative surgery.^43^ An accurate diagnosis ruling out node invasion also might be useful in the choice between elective neck dissection (END) and clinical surveillance.^44^ Preoperative tumor imaging based on computed tomography (CT) scan, magnetic resonance imaging (MRI), and positron emission tomography (PET) scan is used, but its discriminative power is not absolute.^43^ The most recent TNM classification recognizes DOI as a useful parameter, although the discussion is ongoing regarding how it should be combined with imaging and which cutoff would best predict the nodal risk.^45^,^46^

Genomic alterations and genome instability are reported in multiple studies to be associated with nodal invasion.^47^–^49^ In a recent study, Biswas et al.^48^ defined the following set of genomic OSCC features linked to nodal extension: somatic hotspot mutations in *TP53* and *CASP8* genes, rare germline mutations in *BRCA2* and *FAT1* genes, mutations in DNA repair and mitotic G2‐M pathways, and chromosomal instability. Using this information, the authors were able to classify most OSCCs correctly according to nodal metastasis (89% with nodal metastasis vs 80% without nodal metastasis).^48^ Interestingly, cfDNA released by HNSCC was used by other authors to track copy-number alterations (CNAs) and genomic instability with the purpose of predicting their nodal status.^49^ A CNA analysis of cfDNA, expressed as an index had a positive predictive value of 90% for the detection of nodal metastases,^49^ suggesting its possible interest in the clinical setting.

Gene-expression signatures reflecting nodal invasion also have been proposed in this context.^50^–^52^ Mes et al.^52^ reported a 22-gene expression signature for the diagnosis of nodal extension in cT1-T2N0 OSCC. The performance of this signature was analyzed by qPCR and shown to have a negative predictive value (NPV) of 84%, (i.e., close to the NPV required to accurately identify patients for whom END might be superfluous). According to their study, using such a signature may theoretically lead to a two-thirds decrease in END in cT1-T2N0 OSCC, replacing it with surveillance.^52^ More recently, the potential use of miRNA profiling also has been explored. Yi Ping Liu et al.^53^ identified a 4-miRNA expression signature predicting nodal metastasis with an AUC of 0.88, suggesting its potential clinical utility.

Overall, all these studies show that gene expression and miRNA signatures can accurately distinguish N0 from N^+ve^ OSCC. However, the extent to which these signatures are portable under the conditions of a clinical diagnosis remains to be shown (i.e., upon analysis of small biopsy samples obtained before surgery). Prospective studies are required to validate their use and to address, for example, the possibility of choosing surveillance over END based on tumor genomics.

Another potential application of genomics might be in the assessment of tumor surgical resectability. Currently, the assessment of tumor resectability depends on the surgeon’s experience, tumor site and size, previous treatments, and the patient’s operability. This initial step of surgical practice does not take into account tumor biology (i.e., pattern of growth, inflammation, grading).^54^ Using data from TCGA, we reported a three-gene expression signature unrelated to the TNM but associated with the presence of positive SMs in resected tongue tumors.^55^ Use of this signature to examine a set of tumors microscopically showed an invasive growth pattern characterized by the presence of immature cancer cells growing as trabeculae, a pattern that might have been missed by initial tumor imaging.^55^ Based on this study, we suggest that analyzing tumor biology with genomics might help in the assessment of tongue tumor resectability.^55^ Although it is unclear at this stage whether tumor biology also is important for other sites of the oral cavity, we suggest that genomics might potentially be useful in determining the nature and extent of the surgical procedure for locally advanced OSCC.^54^ For example, analyzing the propensity of OSCC to invade bone tissue could be useful in determining the extent of mandible resection in case of bone extension.^54^

## Predicting Postoperative Risk in Locally Advanced Tumors

Locally advanced oral cavity tumors (i.e., large tumors [T3T4] ± node involvement) are the most frequent clinical presentation of OSCC.^1^ The main determinants that guide the choice of postoperative adjuvant therapy are the SM status, the number and size of metastatic lymph nodes, and the presence of ENE.^1^ Other criteria such as the PNI and DOI also are important,^1^,^2^ but assessment of the postoperative risk nevertheless remains challenging. It currently is difficult to predict which patients are most likely to benefit from intense adjuvant therapy or, conversely, from therapeutic de-escalation.

Molecular margin analysis (i.e., assessment of the macroscopically normal area of the mucosa after tumor resection) might improve the pathologic diagnosis.^56^ Various genomic alterations, ranging from single nucleotide mutations to epigenetic alterations, are detected in the histologically normal area of the oral mucosa after an apparently successful surgical resection.^56^ Detection of single nucleotide mutations in *TP53* was for the first time suggested to be useful in the seminal study by Brennan et al.^57^ and later confirmed in an independent study.^58^ Microsatellite alterations also were used to track the origin of local OSCC recurrence with R0-clear SMs.^59^ Molecular margin analysis based on mutation/microsatellite analyses was instrumental in exploring the contribution of residual cancer cells/mucosal fields with preneoplastic changes to local relapse/secondary primary tumors.^60^

The practical application of molecular margin analysis awaits more studies to establish the genomic alterations present in the morphologically normal oral mucosa. The fact that some of these alterations might be detected in wide pre-malignant fields poses questions regarding the optimal sampling protocols to be used. It also complicates the interpretation of molecular SM analysis.^56^

Despite the practical limitations encountered with genomics in the detection of cancer cells within SMs, an increasing number of studies point to the peri-tumoral region as a source of interesting biological information. For example, Liu et al.^61^ analyzed SMs to explore the occurrence of microsatellite instability (MSI) in OSCC. An MSI^+ve^ status was detected in 55% of OSCCs, and it was associated with a sevenfold increased risk of local recurrence during a 2-year follow-up period.^61^ The biological rationale behind this instability currently is unknown considering that the canonical MSI syndromes produced by defective DNA mismatch repair are rarely found in OSCC.^62^ Nevertheless, detecting microsatellite alterations (either in the tumor or in the peri-tumoral area) might be of interest as an adjunct to prediction of the risk for recurrence and potentially the response of OSCC to immune checkpoint blockers.^63^ Other studies analyzing gene expression^64^ or specific patterns of DNA methylation^65^ from SMs found that this information potentially predicted a higher risk of postoperative recurrence in patients with R0-clear margins.

Analysis of the tumor itself also is emerging as a valuable source of information to refine risk prediction. For example, the presence of high-risk *TP53* mutations (identified with the Evolutionary Action (EA) scoring system)^66^ or higher intra-tumor genetic heterogeneity (scored with the mutant allele tumor heterogeneity (MATH) system)^67^ offers exciting perspectives for the identification of aggressive OSCC. The study of PNI provides an example of the power of functional genomics in the assessment of the postoperative risk for OSCC.

**Fig. 3:**
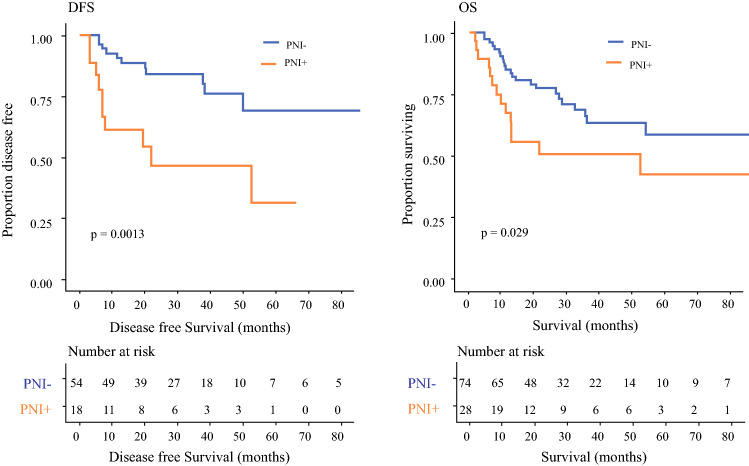
A perineural invasion (PNI) gene expression profile identifies oral squamous cell carcinoma (OSCC) prone to recurrence. Kaplan–Meier analyses of disease-free survival (DFS) and overall survival (OS) in low to intermediate risk OSCC (*n* = 102 from TCGA) are based on the presence or absence of the PNI gene expression profile, as defined in Saidak et al.^70^ Tumors with low to intermediate risk are defined as T1/2 N2 or T3 N0-2, without extracapsular spread or surgical margins (SMs). Patients are divided into positive/negative PNI gene expression profile groups based on the average *z* for 26 PNI genes, with the cutoff at 0

Perineural growth and tumor extension along nerve tracts are a frequent pathologic finding in OSCC.^68^ Although PNI is recognized as an aggressive tumor determinant, its pathologic diagnosis is poorly standardized, and morphologic criteria might not be the most accurate reflection of the underlying biology.^69^ Using gene expression data available from TCGA, we constructed a PNI-associated gene-expression signature that we used for a standardized molecular diagnosis in this cohort.^70^ Interestingly, this PNI signature was found to predict post-surgical recurrence in locally advanced HNSCC in the absence of the strongest risk factors (SM or ENE), raising the interesting possibility that it might be an independent contributor to post-surgical recurrence^70^ (Fig. 3).

The study by Schmidt et al.^71^ further demonstrates the interest of functional genomics for risk assessment in HNSCC by showing that the expression status of only a few genes (*n* = 7) linked to tumor radiobiology, including genes involved in hypoxia-signaling, predicts postoperative recurrence of locally advanced HNSCC treated with radio(chemo)therapy.^71^ Importantly, functional genomics allows the rapid transposition of the most recent research results, from studies exploring genome repair, cellular heterogeneity, cell death regulation, and the like to clinical practice.^72^ The transposition of these tools to the clinic nevertheless awaits validation in independent cohorts and prospective clinical trials.

## Measuring Minimal Residual Disease and Optimizing the Longitudinal Monitoring of OSCC

Despite the use of curative treatments, locally advanced OSCCs frequently recur either locally or at distant sites.^1^ The concept of measuring minimal residual disease (MRD) was first popularized in oncohematology as a strategy to anticipate cancer recurrence by tracking histologically undetectable cancer cells. Mutiple minimally invasive analyses commonly denoted as “liquid biopsies” could be used in this context. In this review, we do not examine the approaches being developed based on the isolation and analysis of circulating tumor cells (CTC) and exosomes.^73^,^74^ Instead, we focus on analyzing cfDNA/RNA shed by tumors as a strategy raising considerable interest for MRD/longitudinal monitoring of OSCC due to the extreme sensitivity of molecular biology techniques.^75^,^76^

Compared with the analyses of conventional biopsies, liquid biopsies based on cfDNA/RNA detection are non-invasive and permit repeated sampling during the course of the disease. They also may reflect the cellular heterogeneity of the tumor more accurately than conventional biopsies.^76^

The practical approaches used to analyze cfDNA shed from tumors can be classified into targeted or tumor-agnostic approaches depending on prior knowledge of the tumor mutational status.^76^ The study by Wang et al.^77^ was the first to establish the principle of cfDNA detection by NGS in HNSCC. Using targeted analysis of only a few genes (*TP53, PIK3CA, CDKN2A, NOTCH1*), the authors were able to establish the tumor “fingerprint” and later track the corresponding mutations of cfDNA in the blood and saliva of 63 HPV-negative HNSCC patients.^77^ Tumor-derived cfDNA was detected in all patients with OSCC (80% of plasma samples and 100% of saliva samples).^77^ Interestingly, cfDNA was detected as early as 19 months before recurrence in the saliva and plasma from one surgically treated patient, providing a proof of principle that sensitive and specific detection of cfDNA is possible in OSCC.^77^

The detection of cfDNA in plasma offers interesting perspectives for the detection of MRD and optimizes longitudinal monitoring of HNSCC. However, the studies reported to date are descriptive and based on the follow-up evaluation of a limited number of patients with different tumor stages and primary locations.^78^,^79^ Nucleic acid analyses of saliva might be more sensitive and better suited for the follow-up evaluation of OSCC in the context of curative therapy, as suggested by the study of Wang et al.^77^ Monitoring postoperative variations in the levels of miRNA in saliva might be useful, as suggested for example with miR-423-5p.^26^

Recently, the prospect of monitoring tumor suppressor gene methylation in oral rinses using a highly sensitive approach of droplet digital PCR also was suggested by Fung et al.^80^ In this interesting study examining DNA methylation of three genes (*PAX5, EDNRB,* and *DCC*) in 50 HNSCC cases (mostly OSCC), the authors observed decreased levels of methylated cfDNA in saliva after surgery. Importantly, a rebound in at least one of the three markers preceded 80% of the local recurrence cases.^80^ Further prospective analyses of tumor cfDNA/RNA in saliva are eagerly awaited considering their possibly superior sensitivity in the monitoring of OSCC.

## Predicting the Efficacy of Medical Treatments

### Therapeutic Targeting of Oncogenic Signaling

Systemic therapies typically are prescribed in the context of recurrent/metastatic HNSCC.^1^,^2^ This review examines the possible application of therapeutic targeting to the perioperative context in OSCC. As recently discussed in detail elsewhere, neoadjuvant treatments do not confer a benefit in terms of survival, but their application for selected patients might be of interest in the context of organ preservation to facilitate tumor resection, or as a way to reduce local or distant recurrence and facilitate treatment deintensification.^81^

Cetuximab, a monoclonal antibody targeting the epidermal growth factor receptor (EGFR), became the first targeted therapy with proven efficacy against HNSCC.^1^,^2^ A recent phase 1b study describes the good tolerability of neoadujant cetuximab given 3–4 weeks before scheduled surgery in HNSCC.^82^ The use of cetuximab, however, illustrates the complexity of therapeutic targeting because its efficacy as a single agent is modest and there are no validated predictive biomarkers.^1^,^2^ Recent progress in understanding EGFR regulation suggests that better therapeutic stratification might be within reach based on analysis of the abundance of EGFR ligand mRNA rather than EGFR itself.^83^

Analysis of somatic mutations and CNA might offer other opportunities.^6^–^8^,^84^ For example, mutations and amplification of the *PIK3CA* gene are present in one-third of OSCCs (Table 1) and provide a rationale for counteracting PI3K/mTOR-signaling.^85^ Findings show that *CCND1* amplification/*CDKN2A* inactivation also are frequent, providing a rationale for targeting regulators of the cell cycle.^83^ However, interpreting genomic alterations remains a complex task, as illustrated by the existence of multiple rare genomic alterations converging on Notch signaling in HNSCC.^86^ Future molecular studies combining genomics with functional assays (e.g., patient-derived cell lines and organoids) and proteomics likely will help to better predict signaling actionability in cancer cells.^87^ At the same time, the re-purposing of “old” drugs offers exciting perspectives for their future clinical use in OSCC. For example, Hedberg et al.^88^ recently observed that nonsteroidal anti-inflammatory drugs (NSAIDs) might confer a significant therapeutic benefit in tumors carrying an activating mutation in the oncogene *PIK3CA.* If these findings are confirmed in a prospective trial, using NSAIDs might be envisioned as a possible adjuvant against OSCC with an active PI3K pathway.

An important application of the analysis of cfDNA shed by OSCC is the detection of targetable genomic alterations.^75^,^76^ Liquid biopsies are ideally suited for this application considering their noninvasive character and the possibility that they might reflect the most active subclone within the tumors.^75^,^76^ Braig et al.^89^ reported the occurrence of somatic mutations activating the RAS genes (*KRAS, HRAS, NRAS*) in the cfDNA of 6 of 13 HNSCC patients receiving cetuximab.^89^ The occurrence of these mutations was correlated with disease progression, suggesting their possible contribution to the emergence of resistance to cetuximab.^89^ Depending on new preclinical findings, new second-line therapeutics might be proposed in this setting.^90^ The integration of liquid biopsies into clinical practice awaits prospective clinical trials examining the use of cfDNA to identify potentially actionable gene alterations.

### Targeting Immune Checkpoints

An important clinical development of the past decade has been the introduction of immune checkpoint blockers (ICBs).^91^,^92^ Nivolumab and pembrolizumab, two monoclonal antibodies targeting the interaction between the molecule PD1 (programmed cell death 1) expressed by immune cells and its receptor PD-L1 (programmed cell death 1-ligand 1, *CD274*) present on the surface of cancer cells, are approved for the treatment of recurrent/metastatic HNSCC.^91^,^92^ To date, PD1-targeting used alone is considered effective for almost 20% of patients with recurrent/metastatic OSCC.^91^,^92^

Importantly, the prospect of applying neoadjuvant immunotherapy is raising considerable interest.^93^,^94^ For primary tumors other than HNSCC, a single dose of immunotherapy can be safely administered before surgery. The presence of the tumor might promote the efficacy of ICBs, perhaps by supplying tumor antigens in the context of immune normalization.^93^,^94^ In practice and in the context of OSCC, neoadjuvant ICBs might also be helpful for tumor-debulking, eventually facilitating surgical resection and a reduction in adjuvant therapy.

Two recent phase 2 trials examined the use of pembrolizumab^95^ or nivolumab, either alone or in combination with ipilimumab (an ICB targeting cytotoxic T-lymphocyte-associated protein 4 [CTLA-4])^96^ in the neoadjuvant context in OSCC. In both studies, a limited number of patients were recruited (22 and 29 OSCC patients, respectively), and the follow-up period did not exceed 1 year.^95^,^96^ Objective responses were found at an appreciable frequency, leading to tumor downstaging of more than half of OSCC cases in the study by Schoenfeld et al.^96^ Neoadjuvant immunotherapy, administered as one or two cycles 3 weeks before surgery, appears to be safe in OSCC.^95^,^96^ These two studies challenge the current surgical practice of proposing a rapid upfront resection of OSCC and emphasize the need to reliably predict the efficacy of ICBs for surgical patients. The immunohistochemical detection of PD-L1 is the only approved analysis in this setting, but this analysis has limited predictive potential and poses problems in terms of standardization.^97^ A number of genomic analyses could be of predictive interest in this setting including the occurrence of somatic *CD274/PD*-*L1* gene amplification,^98^ the detection of mismatch repair deficiency and the tumor mutational load,^99^ and transcriptional signatures that unveil the presence of an active immune cell infiltrate (T cells, M1 macrophages, and the expression of immune checkpoint molecules other than PD-L1).^100^,^101^ This list is expanding as novel actors within the tumor microenvironment that modulate the tumor response to ICBs are identified, such as B cells or myeloid-derived suppressor cells.^102^

Interestingly, many facets of immunoregulation are amenable to genomics, such as hypoxia/tumor metabolism or microbial metagenomics.^102^ Clonal structure, reconstructed from tumor sequencing, also might be interesting.^67^ Besides the difficulty predicting their efficacy, ICBs challenge the usual procedures used for the follow-up evaluation of OSCC patients, as illustrated by the pseudo-progression that can be observed in some patients.^91^ To monitor the response of solid tumors treated by ICBs, cfDNA monitoring could be useful,^103^,^104^ but no studies to date have reported its use in OSCC.

Importantly, an increasing number of patients with recurrent/metastatic OSCC receive concomitant ICBs and radio(chemo)therapy. Several ongoing trials are exploring the existence of possible synergies between “conventional” treatments, particularly radiotherapy, and ICB.^105^–^107^ Genomics not only may identify biomarkers of individual sensitivity to ICBs, but also may bring a rationale for their combination with radio(chemo)therapy.^105^–^107^ This exciting prospect calls for a reexamination of the biological determinants of the OSCC response to radio(chemo)therapy beyond the criteria of local control/survival. For example, predicting OSCC sensitivity to apoptosis induced by radio(chemo)therapy might be useful for the identification of tumors likely to benefit from adjuvant protocols combining conventional therapies and ICBs. This research has the potential to change current medical practice for recurrent/metastatic OSCC. It also illustrates how genomics is well-suited to address the complex biology behind tumor response to ICBs.

## Perspectives and Conclusion

Although DNA/RNA sequencing has become widespread.^10^,^11^ It still is not used in clinical practice for OSCC.^1^ As can be seen from our overview of its potential clinical applications (Table 2), genomics is a powerful research tool that has numerous potential clinical applications. A popular application of genomics lies in the definition of homogeneous cohorts of patients before their recruitment in clinical trials, a key step for testing medical treatments against tumors at advanced stages.^108^ We anticipate that the clinical application of genomics will expand in the coming years to earlier “surgical” stages of OSCC. Nevertheless, a number of practical challenges remain before genomics can be routinely used in this setting (Table 3). Further research also is necessary to unfold the complex biology of oral tumor development and transform oral oncology into precision medicine/surgery. In the future, exploration of the non-coding genome, the epigenome, and molecular events at a single-cell resolution with cfDNA/RNAl likely will be a source of new minimally invasive biomarkers.

**Table 2:** An overview of genomics applied to OSCC

Application	Molecule analyzed	Analysis/genomic region examined	Source	References
Oral potentially malignant lesions, early diagnosis of OSCC	DNA	Loss of heterozygosity (3p14 + 9p21)	Lesion	15
	DNA	Loss of heterozygosity + *TP53* mutation	Lesion-brushing	16
	mRNA	Microarray, pan-genome expression analysis	Lesion	17
	miRNA	qPCR	Oral rinses, saliva	25,26
	Methylated DNA	MS-qPCR, targeted signatures (3 to 13 genes)	Lesion-brushing, saliva	34–38
	Methylated DNA	cfDNA NGS, over 1 million methylated CpGs	Plasma	39
Tumor staging before surgery	DNA	FISH, *CCND1* amplification	Tumor	47
	DNA	NGS, mutations (somatic & germinal) + CNA	Tumor	48
	DNA	cfDNA NGS, whole genome/shallow sequencing, CNA	Plasma	49
	mRNA	microarray, qPCR, RNA seq NGS	Tumor	50–52,55
	miRNA	RNA seq NGS	Tumor	53
Predicting postoperative risk	DNA	PCR + Sanger sequencing, missense mutations in TP53	Surgical margins	57,58
	DNA	Microsatellite instability	Surgical margins	61
	mRNA	Nanostring/4-gene signature	Surgical margins	64
	Methylated DNA	Quantitative methylation-specific PCR	Surgical margins	65
	DNA	NGS: missense mutations TP53 (EA score), intratumoral heterogeneity (MATH)	Tumor	66,67
	mRNA	NGS/pan-genome expression analysis: PNI signature	Tumor	70
	mRNA	Nanostring/7-gene expression signature	Tumor	71
Minimal residual disease & longitudinal monitoring	DNA	cfDNA NGS, targeted sequencing *(TP53*, *PIK3CA*, *CDKN2A*, *FBXW7*, *HRAS*, and *NRAS)*	Saliva, plasma	77
	DNA	cfDNA NGS, whole-exome sequencing. droplet digital PCR	Plasma	78,79
	miRNA	qPCR (miR-423-5p)	Saliva	26
	Methylated DNA	Digital droplet MS-qPCR/gene-methylation (*PAX5, EDNRB, DCC*)	Oral rinses	80
Predicting the efficacy of medical treatments	DNA	NGS, whole-exome sequencing (*PIK3CA* mutation)– NSAIDs	Tumor	88
	DNA	NGS, targeted sequencing (*HRAS, KRAS, NRAS*)–cetuximab	Tumor + plasma	89
	DNA	FISH, *CD274*/PD-L1 amplification	Tumor	98
	DNA + mRNA	NGS, whole-genome analysis (microsatellite instability, Tumor mutational burden, T cell infiltration and cytokine expression profile)	Tumor	99
	DNA + mRNA	NGS, pan-genome expression analysis	Tumor	100
	mRNA	qPCR, (panel of 46 immune genes)	Tumor	101

OSCC, oral squamous cell carcinoma; qPCR, quantitative polymerase chain reaction; MS-qPCR, methylation-specific qPCR; cfDNA, cell-free DNA; NGS, next-generation sequencing; FISH, fluorescence in situ hybridization; CNA, copy number alteration; cfDNA, cell-free DNA; EA score, evolutionary action score; MATH score, mutant allele tumor heterogeneity score; NSAIDs, non-steroidal anti-inflammatory drugs

**Table 3:** Some of the challenges ahead for practical application of genomics to OSCC

Challenges	Proposed solutions
Variety of analytical platforms and strategies used.	Quality programs + cross-laboratory concordance assays
Data complexity: new parameters (gene expression profiles, rare variants and mutations, tumor mutational burden, tumor structure and clonality), necessity to integrate with clinical, radiological and histopathological data	Computer approaches and specialized expertise for data-handling and integrative analysis
Cost & investments required	Technical development/rationalization of the implementation
Facilitate translational research and clinical trials	Organization of large collections, with well-annotated material, develop open access

OSCC, oral squamous cell carcinoma
